# Supplementary material for: Transitioning from global to local computational strategies during brain-machine interface learning
Source: Front Neurosci. 2024 Apr 19;18:1371107. doi: 10.3389/fnins.2024.1371107 (PMC11066153; doi:10.3389/fnins.2024.1371107)
Supplement: Supplementary file 1 [file Image_1.pdf]

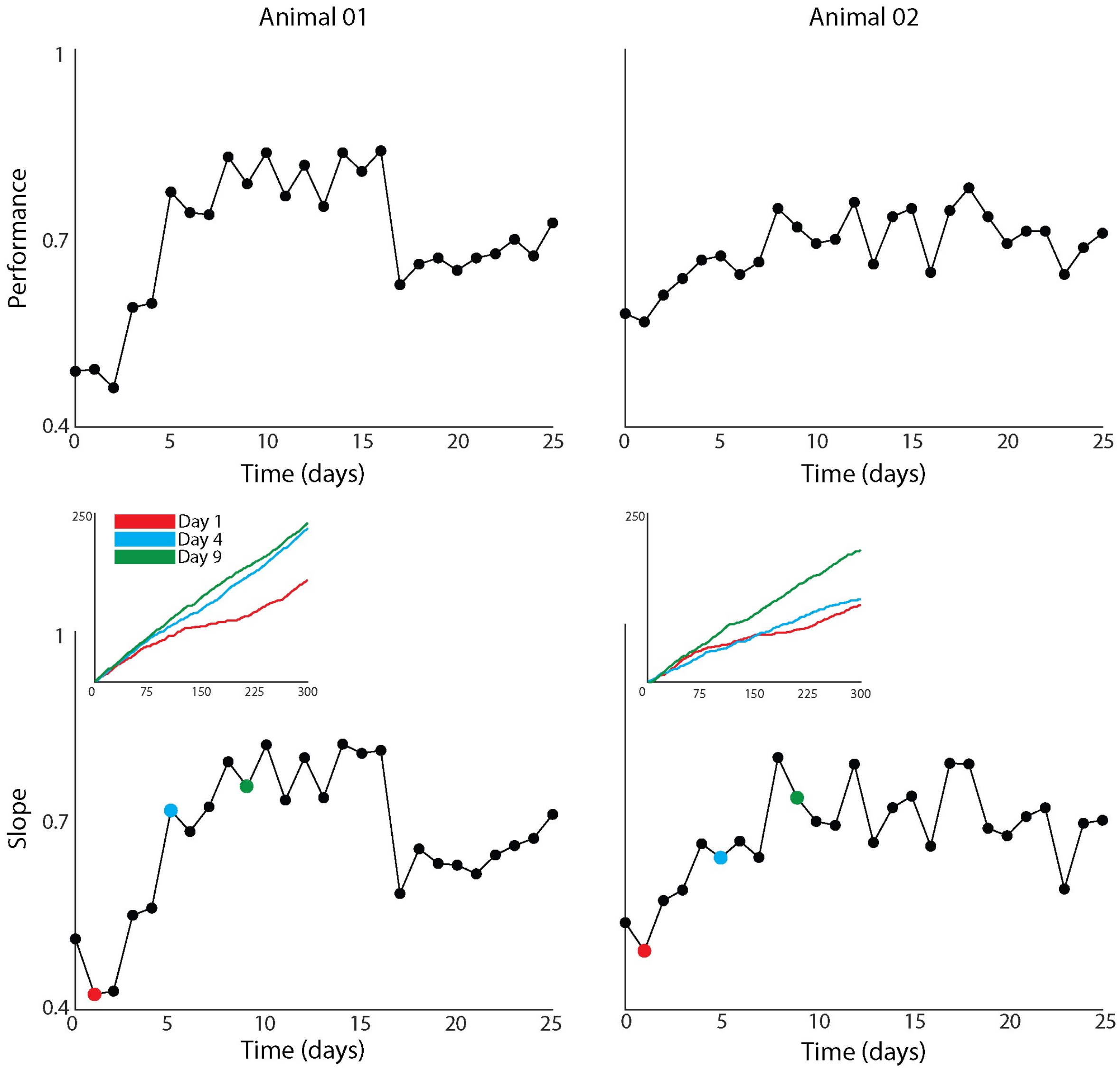

Supplemental Figure 1: Examples showing the performance (top panels) and the slope of the cumulative sum of performance within a recording session (bottom panels) for two animals. Insets for bottom panels show the cumulative sum of the current trials during single recording sessions (Day 1, 4 and 9 as examples). From the insets, it is clear the cumulative sum of the correct trials is linear, also see text. The slope of the cumulative sum for each day a measure of how much was learned, the steeper the slope the more correct trials during that recording session. The slopes increase from earlier days (e.g. Day 1) to later days (e.g. Day 9). When the slope of the cumulative sum is plotted for each day (bottom panels) the shape of the curves are similar to that animal's performance (top panels), suggesting that the rate at which animals learn during a session is well described by the overall performance of that session.
